# Low-Thermal-Budget Photonic Sintering of Hybrid Pastes Containing Submicron/Nano CuO/Cu_2_O Particles

**DOI:** 10.3390/nano11071864

**Published:** 2021-07-20

**Authors:** Po-Hsiang Chiu, Wei-Han Cheng, Ming-Tsang Lee, Kiyokazu Yasuda, Jenn-Ming Song

**Affiliations:** 1 Department of Materials Science and Engineering, National Chung Hsing University, Taichung 402, Taiwan; pohsiangchiu@smail.nchu.edu.tw (P.-H.C.); g108066021@mail.nchu.edu.tw (W.-H.C.); 2 Department of Power Mechanical Engineering, National Tsing Hua University, Hsinchu 300, Taiwan; mtlee@pme.nthu.edu.tw; 3 Division of Materials and Manufacturing Science, Graduate School of Engineering, Osaka University, Osaka 565-0871, Japan; yasuda@mapse.eng.osaka-u.ac.jp; 4 Innovation and Development Center of Sustainable Agriculture, National Chung Hsing University, Taichung 402, Taiwan

**Keywords:** photonic sintering, copper oxide, copper salts, hybrid paste

## Abstract

Copper oxide particles of various sizes and constituent phases were used to form conductive circuits by means of photonic sintering. With the assistance of extremely low-energy-density xenon flash pulses (1.34 J/cm^2^), a mixture of nano/submicron copper oxide particles can be reduced in several seconds to form electrical conductive copper films or circuits exhibiting an average thickness of 6 μm without damaging the underlying polymeric substrate, which is quite unique compared to commercial nano-CuO inks whose sintered structure is usually 1 μm or less. A mixture of submicron/nano copper oxide particles with a weight ratio of 3:1 and increasing the fraction of Cu_2_O in the copper oxide both decrease the electrical resistivity of the reduced copper. Adding copper formate further improved the continuity of interconnects and, thereby, the electrical conductance. Exposure to three-pulse low-energy-density flashes yields an electrical resistivity of 64.6 μΩ·cm. This study not only shed the possibility to use heat-vulnerate polymers as substrate materials benefiting from extremely low-energy light sources, but also achieved photonic-sintered thick copper films through the adoption of submicron copper oxide particles.

## 1. Introduction

An important trend in microelectronics is the manufacture of conductive circuits, interconnections, and joints at low processing temperatures by metallic NPs and their low sintering temperature. The surfactants of NPs need be desorbed, and the NPs coalesce to achieve conductive networks with favorable mechanical strength and electrical conductance [1,2,3,4,5].

On account of the increasing demand for circuits on flexible and stretchable substrates, nanoparticle sintering under electromagnetic irradiation, for example, lasers, near-infrared radiation, or pulsed flashes, has attracted much attention [6,7,8]. In flash light sintering, for instance, the absorption of light by nanoparticles via surface plasmon resonance generates heat. A drastic rise in temperature can evaporate organic surfactants on NPs, induce localized melting, and thereby cause necking among the particles. However, excess irradiation energy may damage polymer substrates.

Kim et al. proposed the photonic sintering of Cu NPs to reduce the material cost [9]. Exposing Cu NPs to intense pulsed xenon flash of an energy density from 20 to 50 J/cm^2^ enables a low electrical resistivity of 5 μΩ·cm to be attained. Two-step flash light sintering with preheating and subsequent main sintering has been proposed to prevent warping of polymer substrate [10]. To improve particle coalescence, a mixture of Cu nanoparticles (20~50 nm in diameter) and microparticles (2 mm in diameter) with an optimal weight ratio of 1:1 has been suggested [11]. However, Cu NPs are easily oxidized and difficult to preserve [12]. To solve this problem, in a revolutionary step, CuO nanoparticles replaced pure Cu NPs in some instances of conductor fabrication by photonic sintering with intense pulse light or lasers [13,14,15,16]. In this process, reducing solvents are required, and the energy of the electromagnetic irradiation causes photochemical reduction, which drives the CuO-to-Cu transformation and the coalescence of particles into continuous networks; this process is typically described by percolation theory.

The fabrication of conductive tracks using copper ion inks has also been suggested. The use of copper formate, acetate and oleate, as well as copper hydroxide, has been proposed to form printed circuits using pulsed light [17,18]. Motivated by these ideas, hybrid pastes that comprise a mixture of submicron and nano-sized copper oxide particles, as well as copper salt additives, are developed herein. This work is the first to explore the effect of copper oxide phase (CuO and Cu_2_O) on the performance of photonic-sintered structure.

## 2. Experimental Procedures

**Figure 1:**
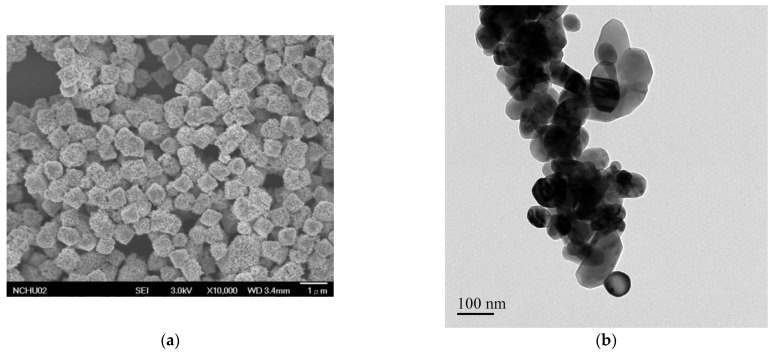
Morphologies of copper oxide particles: (**a**) SEM image of submicron copper oxide particles (SMPO), and (**b**) TEM image of copper oxide nanoparticles (NPO).

**Figure 2:**
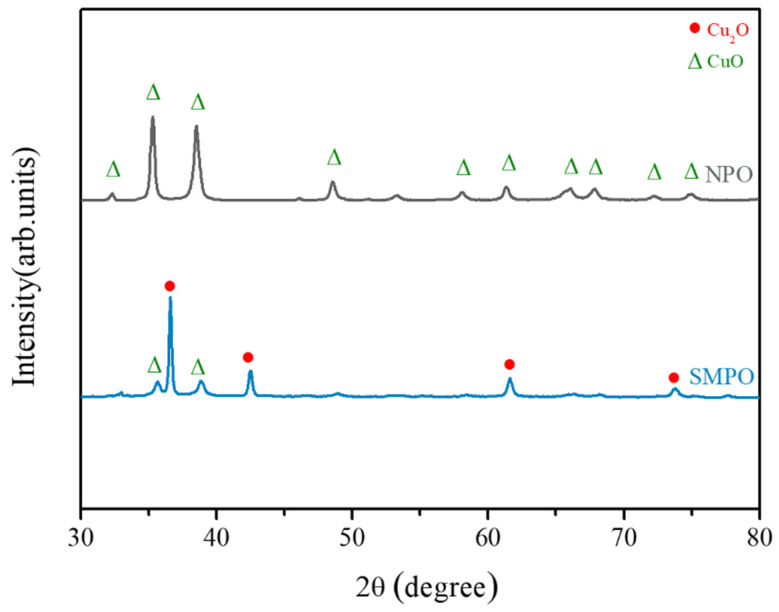
XRD results for submicron copper oxide particles (SMPO) and copper oxide nanoparticles (NPO).

Commercially available submicron copper oxide particles (SMPO, with a diameter of about 500~600 nm, Figure 1a), and CuO nanoparticles (NPO, with an average diameter of approximately 100 nm, Figure 1b), were used herein. XRD phase identification, as presented in Figure 2, shows that SMPO consisted of Cu_2_O and CuO, and NPO comprised merely CuO.

Hybrid pastes were developed by mixing SMPO, NPO, α-terpineol as the solvent, and polyvinylpyrrolidone (PVP, Mw55000, Sigma Aldrich, Burlington, VT, USA) as the paste thickener. The paste was 60 wt.% oxide particles, 34 wt.% α-terpineol, and 6 wt.% PVP. Copper salt additive effects on performance of sintered structure were investigated. The copper salts were cupric sulfate (CuSO_4_∙5H_2_O, 99%; Shimakyu’s pure chemical, Osaka, Japan), copper acetate (Cu(CH_3_COO)_2_∙H_2_O; Shimakyu’s pure chemical, Osaka, Japan), copper(II)formate ((HCO_2_)_2_Cu, Alfa Aesar, Ward Hill, MA, USA), and cupric chloride anhydrous (CuCl_2_; Choneye pure chemical). Some pastes were formed by adding 10 wt.% formic acid to promote the reduction of the copper oxides. The developed pastes were stencil-printed on polyimide films, dried by near-infrared radiation, then sintered using pulsed xenon flashes in ambient atmosphere. The power density of NIR was 3.53 W/cm^2^ and that for xenon flash light was 4.7 W/cm^2^. The flash pulses had an on-time of 0.52 ms and an off-time of 0.33 s. Energy density was estimated to be 1.34 J/cm^2^.

## 3. Results and Discussion

### 3.1. Optimal SMP/NPO Ratio for Hybrid Pastes

**Figure 3:**
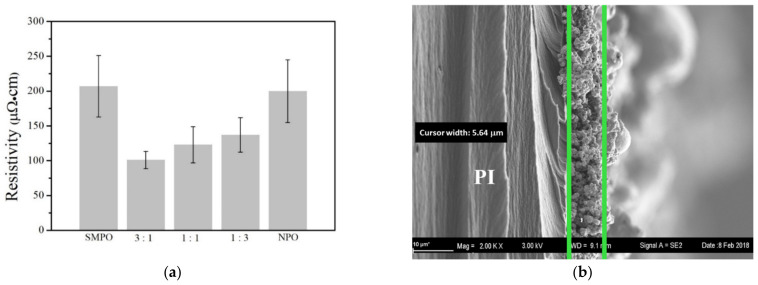
(**a**) Electrical resistivity of sintered structure with various SMPO/NPO weight ratios, and (**b**) cross-sectional image of sintered SMPO samples. Three flash pulses were used to NPO, and four were used to the other samples.

Figure 3a plots the electrical resistivity of the sintered structures with various weight ratios of SMPO to NPO ratios under three or four flash pulses. SMPO/NPO mixed structures had much lower resistivity than those that comprised merely single-sized copper oxide particles. As shown, the sintered SMPO structure had an electrical resistivity of 207.5 ± 44.1 µΩ·cm, while that of the sintered NP structure was 199 ± 45 µΩ·cm. Mixing NPO with SMPO improved electrical conductance. The lowest resistivity, 101 ± 12 µΩ·cm, was achieved with a mixture of SMPO:NPO = 3:1. The sintered structures possessed average thickness of about 6 μm based on the SEM cross-sectional images. For instance, Figure 3b reveals that the thickness of sintered SMPO was 5.6 μm.

**Figure 4:**
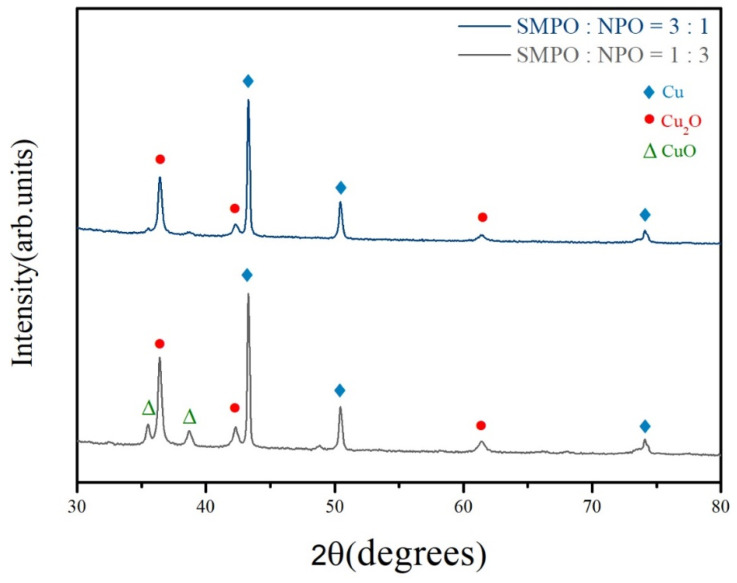
XRD patterns of hybrid pastes with various SMPO/NPO mixing ratios exposed to xenon flash pulses.

According to the XRD patterns in Figure 4, the major constituent phase of the sintered structures was pure copper. Cu_2_O and CuO were identified in the SMPO:NPO = 1:3 samples, but in the SMPO:NPO = 3:1 samples, Cu_2_O was the only oxide detected. The above results suggest that SMPO:NPO = 3:1 is the optimal ratio for obtaining the flash-sintered copper structure with least amount of residual oxides and lowest electrical resistivity.

### 3.2. Effect of Copper Salts on Photonic Sintering of Copper Oxides

**Figure 5:**
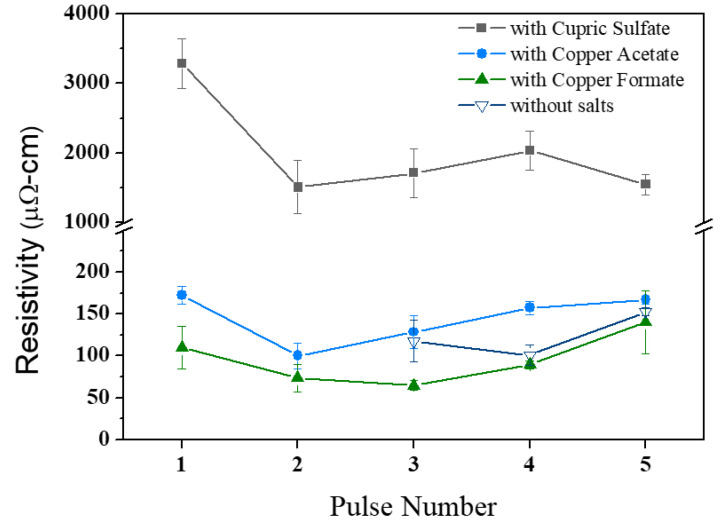
Resistivity of sintered hybrid copper pastes with various copper salt additives (and SMPO:NPO ratio of 3:1).

**Figure 6:**
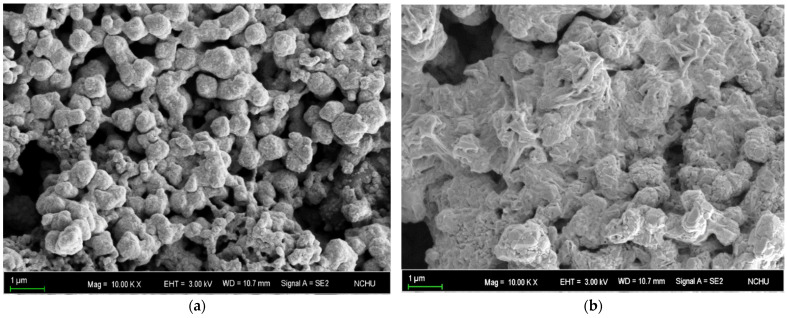
SEM image of sintered hybrid copper pastes (**a**) without copper salts, and (**b**) with copper formate.

Figure 5 plots the electrical resistivities of flash-sintered structure of hybrid pastes with various copper salt additives, i.e., cupric sulfate, cupric chloride, copper formate, and copper acetate. Formic acid was added to 10 wt.% to promote oxide reduction, and one-quarter of the copper oxides was replaced by copper salts to form salt-bearing pastes. Pastes that does not contain salts had a resistivity of around 100 µΩ·cm. Of the copper salt additives, copper formate was the only one that reduced electrical resistance. Exposure to three flash pulses reduced the electrical resistivity to 64.6 ± 5.7 µΩ·cm. Adding copper acetate reduced the critical number of flash pulses from four to two, but it did not significantly affect the electrical conductance (~100 µΩ·cm). Adding copper sulfate and chloride contributed to negative effect on electrical conductance. The cupric chloride-bearing sintered structure had an extremely high electrical resistivity, which was beyond the range of interest. Figure 6 presents the microstructural morphologies of the sintered hybrid pastes with or without copper formate. By means of comparison, copper formate promoted the consolidation of the particles and thereby improved both the microstructural continuity and electrical conductance.

**Figure 7:**
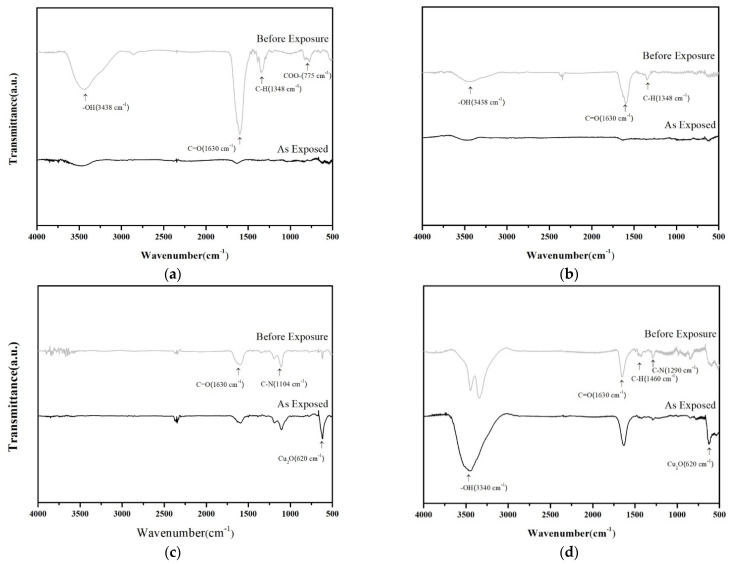
FTIR spectra of sintered structures obtained from hybrid pastes with various copper salts, and numbers of the flashes: (**a**) copper formate with four pulses, (**b**) copper acetate with three pulses, (**c**) cupric sulfate with three pulses, (**d**) cupric chloride with four pulses.

FTIR analyzed the sintered structure and detected copper salt residues; the obtained spectra are shown in Figure 7. Prior to irradiation (the spectrum “before exposure” in each figure), various organic ligands were identified. In order of decreasing wavenumber, they were ν_O-H_ (3438 cm^−1^) from α-terpinol, ν_C=O_ (1630 cm^−1^) from PVP or formic acid, δ_C-H_ (1348 cm^−1^) from PVP or α-terpinol, and ν_C-N_ (1230 cm^−1^) from PVP. Some other ligands associated with particular pastes were also identified, including δ_COO-_ (775 cm^−1^) from copper formate and ν_S=O_ (1415~1350 cm^−1^) from cupric sulfate. Flashing at the pastes that contained carboxylates (copper formate and copper acetate) almost eliminated the organic ligands. In contrast, the ligand signals of ν_C-N_, ν_C=O_, and δ_C-H_ remained detectable from pastes with cupric sulfate and cupric chloride. The ν_O-H_ signal in cupric chloride-containing pastes was almost unaffected. This result reveals that carboxylate was relatively readily decomposed under pulsed flash irradiation, and converted into metallic copper, favoring to conductive path linking.

**Figure 8:**
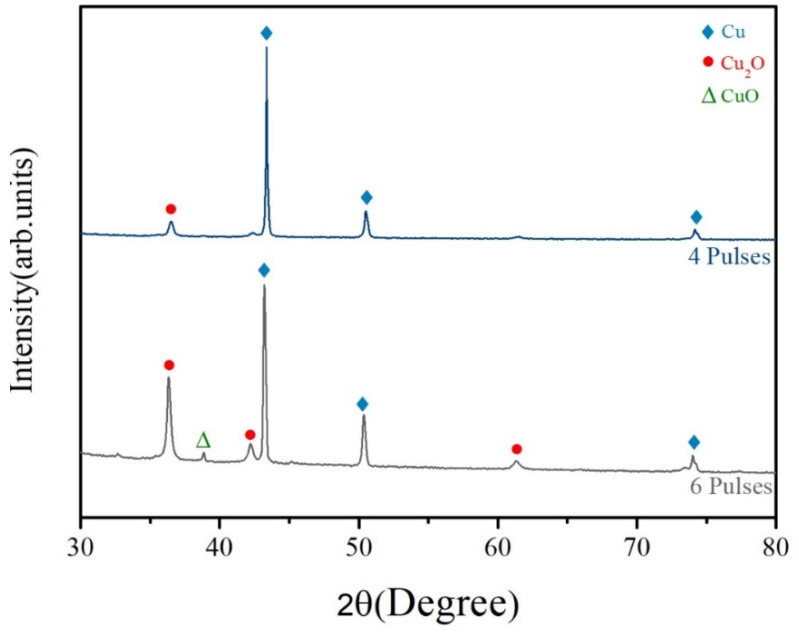
XRD results for hybrid copper pastes that contain copper formate following flash pulses.

As also illustrated in Figure 5, when hybrid pastes containing copper formate were exposed to three flash pulses, their electrical resistivity achieved its lowest value, after which it increased gradually. Figure 8 displays the XRD patterns of sintered structures that were collected from copper formate-containing pastes that were exposed to four or six flash pulses. Cu_2_O diffraction peaks became intensified and that of CuO re-emerged as the number of pulses increased from four to six. This result indicates that excess flash pulses caused oxidation of the reduced copper and thereby worsened electrical conductivity.

### 3.3. Mechanisms and Factors That Affect Electrical Conductance of Flash-Sintered Structures of Copper Oxide Particles

**Figure 9:**
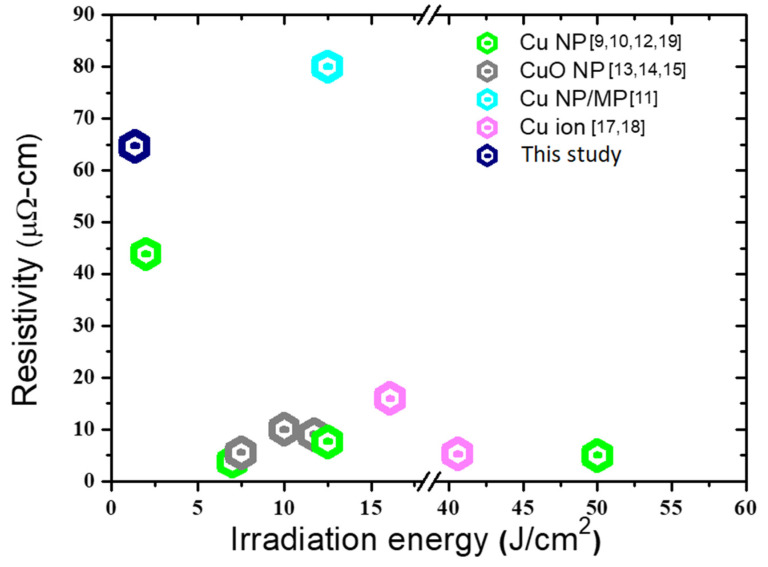
Materials (copper/copper oxide), flash irradiation energy and electrical resistivity of photonic-sintered structures from the literature and in this study.

Figure 9 compares the electrical resistivities of various Cu/CuO pastes that were subjected to flashes with various irradiation energies. Although the electrical resistivity that was obtained herein exceeded most reported values, it still fulfilled the requirement for acceptable electrical resistivity, i.e., 100 μΩ·cm [15]. Pulsed flashes with an extremely low energy density were used in this study to prevent damages to vulnerable substrates while providing acceptable electrical conductance of the sintered structure. The used of a mixture of submicron-nano-sized copper oxide powders is proposed here for the first time on account of its favorable cost, ease of storage, and relatively long shelf-life. Worth of notice is that nano-Cu or CuO inks usually form sintered structure with the thickness of 1 μm of less. The sintered films of hybrid pastes reached 6 μm in average thickness. This might account for the inferior electrical conductance but shed the likelihood to fabricate thick copper films.

The mechanism of the reduction of copper oxide into pure copper by pulsed flash lights has seldom been studied and is therefore not yet sufficiently understood. The reactive sintering reaction that was suggested by Ryu et al. [8] can be used in this process. PVP, which was adopted as paste thickener to adjust the paste viscosity, is a kind of photoactive polymer, which may decompose when exposed to irradiation, forming intermediate acids or alcohol that reduce copper oxides (CuO and Cu_2_O) as following steps.

5CuO + CH_3_COOH → 3Cu + Cu_2_O + 2H_2_O + 2CO_2_ (1)

4Cu_2_O + CH_3_COOH → 8Cu + 2H_2_O + 2CO_2_ (2)

Photo-decomposition of α-terpineol and PVP may form not only acids or alcohol but also OH radicals [19,20], which may also promote the reduction of copper oxide to pure copper during the xenon flash pulse process via reactions (3) and (4).

CuO + 2OH → Cu + H_2_O + O_2_ (3)

2CuO + 2OH → Cu_2_O + H_2_O + O_2_ (4)

**Figure 10:**
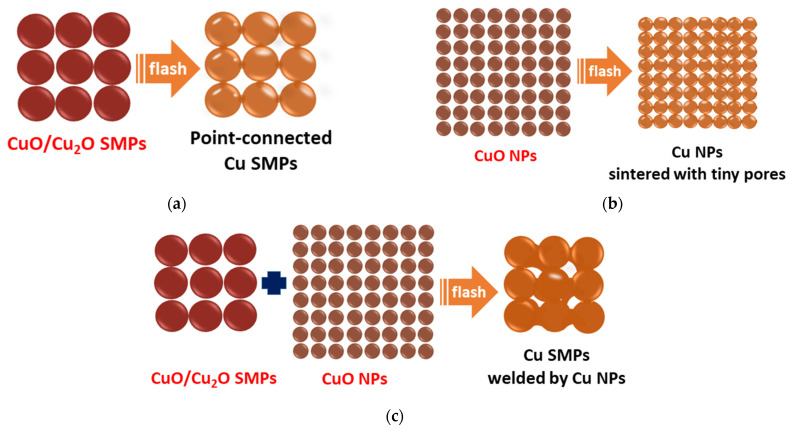

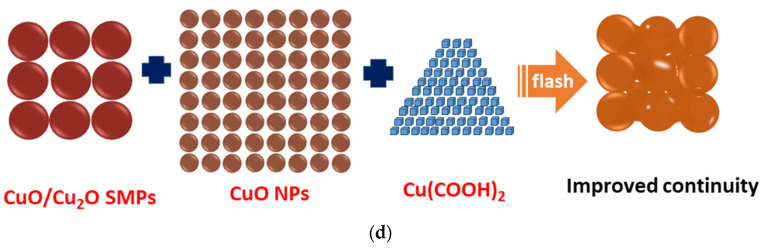
Schematics showing paste ingredients and obtained photonic-sintered structures: (**a**) SMPO only, (**b**) NPO only, (**c**) SMPO and NPO, and (**d**) SMPO, NPO, and copper formate.

The electrical resistivity variation of sintered structures with the SMPO/NPO ratio is associated with the particle size and constituent phases. Mixing Cu or Ag particles of various sizes and shapes has been proposed to improve the linking of sintered structures [7,21]. Mixing copper oxide particles of various sizes is herein proposed for the first time. As shown in Figure 10a, the sintering of submicron-sized SMPO left interstices, forming a loose structure and thereby, inferior connections among particles. The right amount of nano-sized NPO can fill the interstices of such a porous structure. However, an excess NPO would increase electrical resistivity, probably owing to the tiny nano-sized pores (Figure 10b,c). These results suggest that adding copper formate can fill the gaps, cause the reduced copper to coalesce, improve the continuity of sintered structures, and thereby, their electrical conductance (Figure 10d).

The constituent phases importantly affect the electrical conductance. As described by Equations (1)–(4), the reduction of CuO to pure Cu is more difficult than that of Cu_2_O. Therefore, the optimal SMPO/NPO ratio and reduced electrical resistivity of flash-sintered structures are proposed to be related to proportion of Cu_2_O. However, excess exposure to light was demonstrated to cause re-formation of oxide, and especially Cu_2_O in the sintered structure, with a consequent increase in electrical resistivity.

## 4. Conclusions

Hybrid pastes that contain nano- and submicron-sized copper oxide particles, as well as copper formate, were successfully developed for the fabrication of conductors by low-energy-density pulsed flash sintering. Unlike the films formed by commercial nano-CuO inks whose thickness is usually about 1 μm or less, several flash pulses can transform this hybrid paste into a conducting sintered copper structure with average thickness of 6 μm. An optimum SMPO:NPO ratio of 3:1 to yield reduced electrical resistivity is suggested. Adding copper formate can lower the resistivity to 64.6 ± 5.7 µΩ·cm. FTIR spectra demonstrate that unlike cupric sulfide and chloride, copper formate can be completely dissociated by flash irradiation. One of the products of salt decomposition, metallic copper, improves structural consolidation and electrical conductance.
